# Epigenetic Signatures as Predictors of Fracture Non-union: A Systematic Review

**DOI:** 10.7759/cureus.115065

**Published:** 2026-08-24

**Authors:** Joseph Salem-Hernández, Emmanuel Belardo, Peter A Santiago-Gadea, Camilla A Pérez-Vicente, Norman Ramírez

**Affiliations:** 1 Department of Orthopaedic Surgery, Ponce Health Sciences University, Ponce, PRI; 2 School of Medicine, Central University of the Caribbean, Bayamon, PRI

**Keywords:** biomarkers, bone healing, dna methylation, epigenetics, fracture non-union, long non-coding rna, microrna, systematic review

## Abstract

Non-union is a recognized complication of fracture healing that imposes prolonged disability, pain, and repeated surgical intervention. Clinical prediction currently depends on patient and injury characteristics that discriminate poorly at the level of the individual patient, and a molecular marker capable of identifying high-risk patients early would permit intensified surveillance or biological augmentation while intervention remains feasible. Epigenetic mechanisms, comprising DNA methylation, histone modifications, and non-coding RNAs, regulate skeletal repair and have been proposed for this purpose. This systematic review, reported according to the Preferred Reporting Items for Systematic Reviews and Meta-Analyses (PRISMA) 2020 and Synthesis Without Meta-analysis (SWiM) guidelines, synthesized evidence published between 2014 and 2025, distinguishing throughout between markers of established non-union and validated predictors of future non-union.

A structured search of MEDLINE supplemented by Google Scholar and semantic literature retrieval yielded 460 records, of which 159 unique records were screened, 120 full texts were assessed, and 30 studies met the inclusion criteria. Six studies formed the core evidence base: four primary studies and two systematic reviews. Serum lnc_GABARAPL2 was elevated in 45 patients with long bone non-union relative to 45 matched controls, with an area under the receiver operating characteristic curve (AUC) of 0.922 and an adjusted odds ratio of 11.661 (95% CI, 3.794 to 35.840), acting through sequestration of miR-302a-3p to suppress osteogenic differentiation.

Long non-coding RNA ENST00000563492 was downregulated in non-union tissue from 24 patients and promoted osteogenesis and angiogenesis through CDH11 and vascular endothelial growth factor as a competing endogenous RNA for miR-205-5p. Five microRNAs, miR-31a-3p, miR-31a-5p, miR-146a-5p, miR-146b-5p, and miR-223-3p, were upregulated in a rat atrophic non-union model, with peak differential expression at day 14. Systemic inflammation reduced Dnmt3b expression in skeletal progenitors, producing hypomethylation of the Rbpjk promoter and consequent Rbpjk upregulation with impaired differentiation, such that promoter methylation at this locus is protective, and restoration of Dnmt3b rather than demethylation rescued repair. No primary study examined histone modifications or non-canonical non-coding RNA classes in non-union.

A single discrimination estimate exists, derived from a case-control design without external validation, in which serum was obtained from patients already undergoing non-union surgery, and no study sampled human material before the outcome was known. Certainty of evidence was low to very low across all marker classes. The literature therefore identifies biologically plausible candidates and one promising circulating marker but does not establish prospective predictive validity; prospective cohorts with early post-injury sampling, standardized outcome definitions, and stratification by fixation strategy are required.

## Introduction and background

Fracture healing is a coordinated regenerative process involving inflammatory signaling, cellular differentiation, angiogenesis, and tissue remodeling [1]. Despite advances in orthopedic care, 5% to 10% of fractures fail to consolidate and progress to non-union, conventionally defined as failure of consolidation within six to nine months or the absence of progressive radiographic healing across three consecutive months [2]. Non-union produces prolonged disability and pain and generates substantial cost through revision surgery and extended treatment courses. Clinical prediction of non-union currently rests on patient-level risk factors such as smoking, diabetes, advanced age, and nutritional status; on fracture characteristics including anatomical site, severity, comminution, and soft-tissue injury; and on treatment variables including fixation stability and infection. Individually and in combination, these factors discriminate poorly at the level of the individual patient, and a marker measurable early in the healing course that identified patients destined to fail would permit intensified surveillance, earlier revision, or biological augmentation while the window for intervention remains open.

Epigenetic mechanisms, defined as heritable changes in gene expression occurring without alteration of the underlying DNA sequence, regulate skeletal development, bone homeostasis, and fracture repair [3,4]. Three classes are relevant to bone biology. DNA methylation involves the addition of methyl groups to cytosine residues within CpG dinucleotides, short sequences in which a cytosine precedes a guanine, and methylation of a gene promoter typically silences transcription of that gene. The DNA methyltransferases DNMT3A and DNMT3B establish de novo methylation patterns, while DNMT1 maintains them through cell division, and methylation regulates the master osteogenic transcription factors RUNX2 and osterix [5]. Histone modifications are chemical changes to the proteins around which DNA is wound and, by loosening or tightening that packaging, determine which genes remain accessible for transcription; specific marks respond dynamically to mechanical loading relevant to the fracture site [6,7]. MicroRNAs are approximately 22 nucleotides in length and bind target messenger RNAs to induce degradation or translational repression, permitting coordinated control of osteogenesis, chondrogenesis, angiogenesis, and inflammation [8,9]. Long non-coding RNAs exceed 200 nucleotides and regulate gene expression through chromatin remodeling, transcriptional and post-transcriptional mechanisms, and sequestration of microRNAs, a competing endogenous RNA effect that indirectly de-represses the targets of the microRNA concerned [10].

Evidence regarding epigenetic markers in non-union falls into three categories that carry materially different clinical implications, and the distinction between them governs the interpretation of this review. Diagnostic or confirmatory markers are measured once non-union has already been diagnosed, typically in tissue obtained at revision surgery or in blood drawn before that operation. Such measurements establish association but cannot establish temporal precedence, and a difference observed at a failed fracture site may reflect the consequences of failure, including chronic inflammation, fibrous interposition, mechanical instability, and prior surgery, as readily as its cause. Prognostic or predictive markers, by contrast, are measured at or near the index injury before the outcome is known, in a cohort followed to a defined endpoint, and only this design supports a claim of prediction or yields discrimination estimates that are not inflated by case-control sampling. Mechanistic evidence derived from animal models or in vitro systems establishes biological plausibility and identifies therapeutic targets but carries no direct human predictive claim. Conflating these categories substantially overstates translational readiness, and previous accounts of this literature, including our own earlier framing, have done so.

Epigenetic marks nonetheless offer genuine theoretical advantages as prognostic markers. They respond dynamically to inflammation, hypoxia, and mechanical loading, potentially providing real-time information about healing status. They are detectable in accessible compartments, including serum, cell-free DNA, and circulating extracellular vesicles, permitting minimally invasive serial sampling [11]. Unlike static genetic variants, they directly regulate gene expression and integrate cumulative environmental exposure such as smoking, diabetes, and systemic inflammation. Against these advantages stand tissue specificity, temporal variability, absent technical standardization, and the requirement for validation across diverse populations. The primary objective of this systematic review was to identify and characterize reported epigenetic markers in fracture non-union across marker classes, to classify each body of evidence by marker role, to appraise risk of bias and certainty of evidence using instruments appropriate to each study design, to determine what predictive performance has actually been demonstrated and under what design, and to define the studies required to establish clinical utility.

## Review

Materials and methods

Protocol and Registration

This review was conducted according to the Preferred Reporting Items for Systematic Reviews and Meta-Analyses (PRISMA) 2020 guidelines [12] and reported in accordance with the Synthesis Without Meta-analysis (SWiM) guideline for syntheses in which meta-analysis is not undertaken [13]. The protocol was not prospectively registered, reflecting the exploratory nature of this emerging field and the need for iterative refinement of the inclusion criteria during the scoping phase, and this is acknowledged among the limitations.

Search Strategy

A structured literature search was performed in PubMed/MEDLINE and supplemented by Google Scholar and SciSpace, a semantic literature retrieval tool used to broaden recall beyond strict keyword matching. Searches were performed in January 2025 and updated in May 2026. The search combined three concept groups using Boolean operators: epigenetic mechanisms (DNA methylation, histone modification, microRNA, lncRNA, non-coding RNA); fracture and bone healing (fracture healing, bone regeneration, osteogenesis, callus formation); and impaired healing (non-union, delayed union, pseudarthrosis, failed fracture healing). The PubMed string was ((DNA methylation[MeSH] OR histone modification[MeSH] OR epigenetic*[tiab] OR microRNA[MeSH] OR miRNA[tiab] OR "long non-coding RNA"[tiab] OR lncRNA[tiab]) AND (fracture healing[MeSH] OR bone regeneration[MeSH] OR "fracture repair"[tiab]) AND (nonunion[tiab] OR "non-union"[tiab] OR "delayed union"[tiab] OR pseudarthrosis[tiab])) AND (2014/01/01:2025/12/31[pdat]). Backward citation searching of all included studies was performed. Yields by source were PubMed (N = 20), Google Scholar (N = 40), and SciSpace (N = 400), giving 460 records in total. Embase, Web of Science, and Scopus were not searched, and because the majority of records were retrieved through a semantic tool whose query behavior is not reproducible in the sense required by PRISMA, the comprehensiveness of the search constitutes a material limitation that is revisited in the Discussion. Only studies published in the English language were considered.

Eligibility Criteria

Studies were eligible if they examined human patients with fractures, animal models of fracture healing, or bone mesenchymal stromal cells or osteoprogenitor cells derived from fracture patients. The exposure of interest was any measured epigenetic marker, including DNA methylation, histone modifications, microRNAs, long non-coding RNAs, or other non-coding RNA species, compared with normal fracture healing, healthy controls, or baseline measurements. Eligible outcomes were fracture non-union, delayed union, impaired healing, or a surrogate marker of osteogenic capacity. Eligible designs were primary observational cohorts, case-control studies, experimental animal models, in vitro mechanistic studies incorporating clinical samples, and systematic reviews published between January 1, 2014, and December 31, 2025. Studies were excluded if they examined only genetic variants without epigenetic measurement, studied bone disease without a fracture healing outcome, were conference abstracts or editorials without original data, or fell outside the specified date range.

Article Identification and Selection

Records were deduplicated on the basis of DOI, title, and author matching. Two independent reviewers screened titles and abstracts, and the same two reviewers assessed full texts against the eligibility criteria. Disagreements were resolved through discussion and, in cases of persistent disagreement, by consultation with a third review author.

Data Extraction

Two reviewers independently extracted data from the full texts of the included studies using a piloted standardized form, and disagreements were resolved by consensus. Extracted items comprised study identifiers, design and setting, sample size, population and specimen type, epigenetic marker and measurement platform, timing of sampling relative to injury and to outcome ascertainment, fracture characteristics, including anatomical site, classification, and fixation method where reported, outcome definition and method of ascertainment, effect estimates and any discrimination metrics, covariates included in multivariable models, and funding sources and conflicts of interest. Records screened but not meeting the inclusion criteria were used for background citation only and were not subject to formal extraction.

Quality Assessment

Appraisal instruments were matched to study design. Human prognostic factor studies were assessed with the Quality In Prognosis Studies (QUIPS) tool [14] across six domains: study participation, study attrition, prognostic factor measurement, outcome measurement, study confounding, and statistical analysis and reporting. Each domain was rated as low, moderate, or high risk of bias. Animal experiments were assessed with the Systematic Review Centre for Laboratory Animal Experimentation (SYRCLE) risk of bias tool [15], and the two included systematic reviews were assessed with A Measurement Tool to Assess Systematic Reviews, version 2 (AMSTAR-2) [16], which yields an overall rating of confidence in the results of the review. Two independent reviewers appraised each study, and disagreements were resolved through discussion. 

Synthesis and Certainty Assessment

The prespecified metric of interest was a measure of discrimination for non-union, comprising the area under the receiver operating characteristic curve (AUC) with its confidence interval, sensitivity, and specificity, supplemented by adjusted effect estimates from multivariable models. Studies were grouped for synthesis first by epigenetic marker class and then by marker role and study design, with primary studies and systematic reviews reported separately in order to avoid presenting the same underlying evidence twice. Quantitative pooling was not undertaken because only one included study reported a discrimination estimate, which precludes meta-analysis of predictive performance, and because the included studies differ in species, specimen type, marker class, measurement platform, timing of sampling, and outcome definition. Synthesis was therefore narrative and is reported according to SWiM [13], with the direction of effect determined from the reported comparison and results tabulated by marker class. Funnel plots and statistical tests of asymmetry were inapplicable in the absence of pooled estimates, and reporting bias is addressed narratively. Certainty of evidence was assessed with the Grading of Recommendations Assessment, Development and Evaluation (GRADE) framework adapted for prognostic evidence, across the domains of risk of bias, inconsistency, indirectness, imprecision, and publication bias.

Results

Search Results

**Figure 1:**
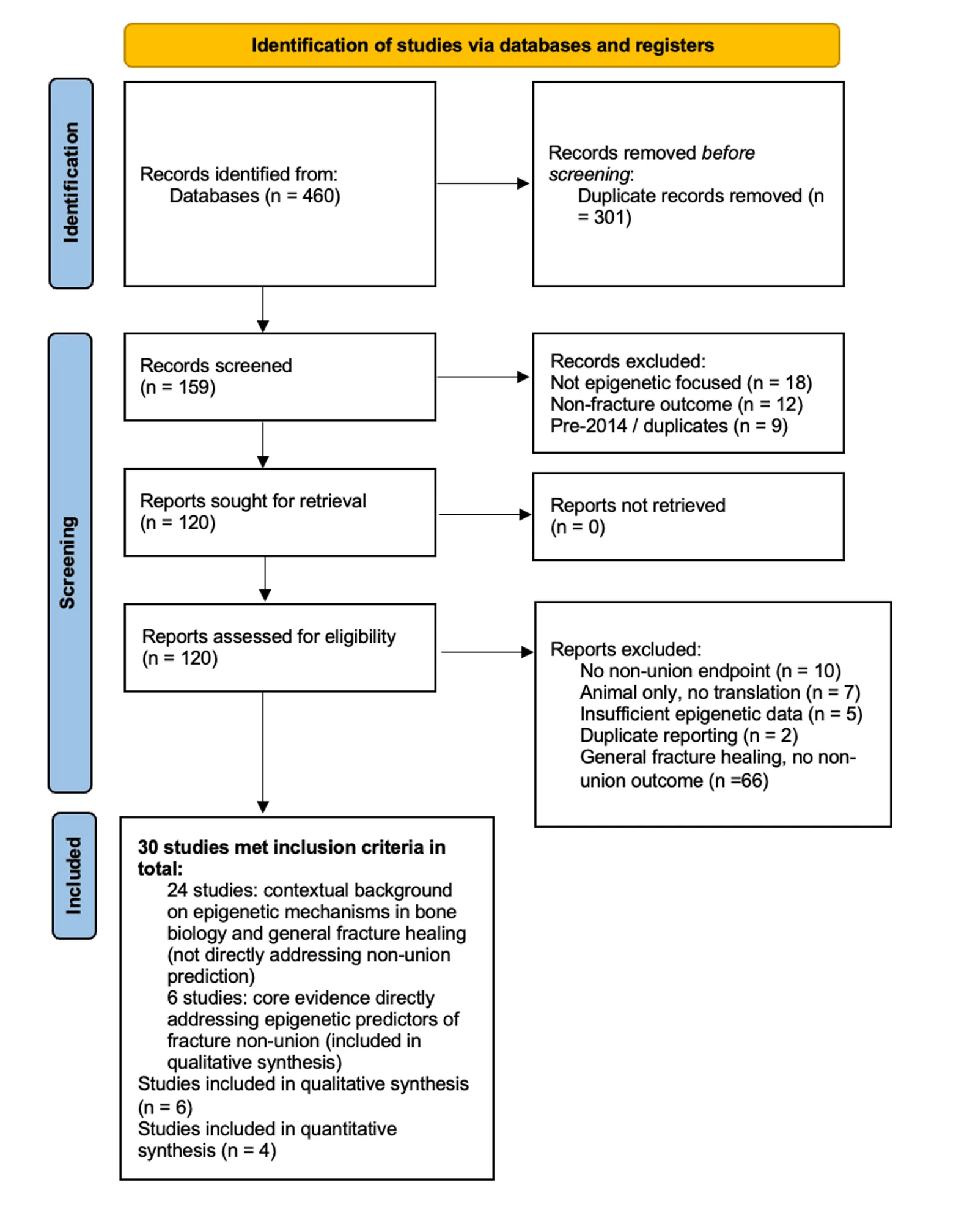
PRISMA 2020 Flow Diagram A total of 460 records were identified (PubMed, N = 20; Google Scholar, N = 40; SciSpace, N = 400). After removal of 301 duplicates, 159 records were screened, and 39 were excluded. Full-text assessment of 120 reports resulted in 90 exclusions, yielding 30 included studies, of which six directly addressed epigenetic markers in fracture non-union and comprised four primary studies and two systematic reviews.

A total of 460 records were identified. After removal of 301 duplicates, 159 unique records underwent title and abstract screening, of which 39 were excluded because they were not epigenetically focused (N = 18), reported no fracture healing outcome (N = 12), or were published outside the eligibility window or were of an ineligible publication type (N = 9). Full texts were assessed for 120 reports, all of which were retrieved. Ninety were excluded, the principal reason being that the study examined general fracture healing without a non-union outcome (N = 66), followed by absence of a non-union endpoint (N = 10), animal-only design without human translation (N = 7), insufficient epigenetic data (N = 5), and duplicate reporting (N = 2). Thirty studies met the inclusion criteria. Six of these directly addressed epigenetic markers in fracture non-union and constituted the core evidence base, comprising four primary studies [17-20] and two systematic reviews [2,11], while 24 provided contextual information on epigenetic mechanisms in bone biology and general fracture healing. The selection process is illustrated in Figure 1.

Study Characteristics

The characteristics of the six core studies are presented in Table 1. Study designs comprised one clinical case-control study with serum sampling and in vitro functional validation [17], one clinical tissue comparison with in vitro and in vivo validation [18], one experimental animal model [19], one experimental study combining single-cell transcriptomics with genetic mouse models [20], and two systematic reviews [2,11]. Studies originated in China (N = 2), Japan (N = 1), Germany (N = 1), the United Kingdom (N = 1), and the United States in collaboration with China (N = 1), and publication activity increased from 2020 onward. Sample sizes, extracted from the full texts rather than the abstracts, were 45 patients with non-union and 45 matched healed controls in Du et al. [17], 10 patients with non-union and 14 with normal healing in Ouyang et al. [18], with RNA sequencing performed on three matched pairs, and 94 rats allocated 1:1 in Waki et al. [19]. Xiao et al. [20] reported no human cohort. With respect to the timing of sampling, serum in Du et al. [17] was drawn preoperatively from patients already scheduled for non-union surgery, tissue in Ouyang et al. [18] was obtained during operation for established non-union, sampling in Waki et al. [19] was serial within a model in which the outcome was determined by the experimental protocol, and Xiao et al. [20] used genetically modified mice throughout. No included study sampled human material before the healing outcome was known.

**Table 1:** Characteristics of Core Studies Directly Addressing Epigenetic Predictors of Fracture Non-union ALP: alkaline phosphatase; AUC: area under the receiver operating characteristic curve; BMP: bone morphogenetic protein; BMSC: bone marrow mesenchymal stromal cell; CI: confidence interval; MSC: mesenchymal stem cell; OCN: osteocalcin; OR: odds ratio; VEGF: vascular endothelial growth factor

Study, Country	Design	Population and Sample Size	Specimen and Marker	Timing Relative to Outcome	Key Findings
Du et al. (2025) [17], China	Clinical case-control with in vitro functional validation; candidate identified in GEO dataset GSE213891	45 long bone non-union and 45 matched healed controls, aged 20 to 50 yr	Preoperative serum; human BMSCs in vitro. lnc_GABARAPL2	Non-union already established at sampling	Elevated in non-union (p < 0.001); AUC 0.922; adjusted OR 11.661 (95% CI, 3.794 to 35.840); suppresses ALP activity, RUNX2, collagen I, OCN, and mineralization; sequesters miR-302a-3p
Ouyang et al. (2020) [18], China	Clinical tissue comparison with in vitro and in vivo validation	10 non-union and 14 normal healing (N = 24); RNA sequencing on 3 matched pairs	Fracture-site scar tissue at operation; BMSCs. lncRNA ENST00000563492	Non-union already established at sampling	Downregulated in non-union; promotes osteogenesis via CDH11 and beta-catenin and angiogenesis via VEGF; competing endogenous RNA for miR-205-5p; enhances bone formation in vivo
Waki et al. (2015) [19], Japan	Experimental animal model	94 rats, 1:1 allocation; femoral healing fracture vs. periosteal cauterization atrophic non-union	Fracture-site tissue, days 3 to 28. miRNA microarray with qPCR validation	Outcome determined by experimental protocol	miR-31a-3p, miR-31a-5p, miR-146a-5p, miR-146b-5p, and miR-223-3p highly upregulated in non-union, peaking at day 14
Xiao et al. (2024) [20], USA and China	Single-cell RNA sequencing with genetic mouse models (Ikk2ca transgenic; Dnmt3b loss of function and transgenic)	Mice; no human cohort	Skeletal progenitor cells. Rbpjk promoter DNA methylation and Dnmt3b	Mechanistic; not applicable	Inflammation reduces Dnmt3b, causing Rbpjk promoter hypomethylation and Rbpjk upregulation with impaired progenitor differentiation; Dnmt3b transgenic mice alleviated the defect
Breulmann et al. (2023) [11], Germany	Systematic review	19 studies on non-union; 82 on general fracture healing	Various. Multiple miRNAs	Mixed	miR-31-5p, miR-221, and miR-451-5p most consistently associated; substantial heterogeneity; clinical utility unvalidated
Panteli et al. (2022) [2], United Kingdom	Systematic review	24 studies of human non-union tissue molecular profiles	Non-union tissue. Various molecular markers including epigenetic factors	Established non-union	Biologically active MSCs present in non-union tissue; altered BMP expression; limited standardized epigenetic data

Quality Assessment

Risk of bias for the six core studies is summarized in Table 2. Among the human prognostic factor studies appraised with QUIPS, the overall risk of bias was moderate in one study and high in one study. In Du et al. [17], prognostic factor measurement was rated low risk on the basis of validated reverse transcription quantitative polymerase chain reaction performed in quadruplicate with documented preanalytical handling, and outcome measurement was rated low risk given radiographic and operative confirmation of non-union. Study confounding was rated moderate because multivariable adjustment included age, sex, American Society of Anesthesiologists classification, injury severity, concomitant injuries, open versus closed fracture, and treatment strategy, but not fracture classification or construct stability. Study participation was rated high risk because a case-control design sampling patients already presenting for non-union surgery cannot support a prospective predictive claim, so that the reported discrimination is a diagnostic rather than a prognostic estimate, and statistical analysis and reporting was rated moderate in the absence of a prespecified analysis plan or external validation cohort. Ouyang et al. [18] was rated high risk overall, driven principally by very small sample size, by RNA sequencing performed on only three matched pairs, and by the absence of any reporting of fixation method, fracture classification, or interval from injury, although the explicit exclusion of patients with infection, tumor, autoimmune disease, pathological fracture, corticosteroid exposure, and smoking history reduces confounding.

**Table 2:** Risk of Bias Assessment of Core Studies AMSTAR-2: A Measurement Tool to Assess Systematic Reviews, version 2; QUIPS: Quality In Prognosis Studies; SYRCLE: Systematic Review Centre for Laboratory Animal Experimentation

Study	Instrument	Principal Concerns	Overall Rating
Du et al. (2025) [17]	QUIPS	Study participation (case-control sampling of patients already presenting for non-union surgery); residual confounding by fracture classification and construct stability; no prespecified analysis plan or external validation	Moderate
Ouyang et al. (2020) [18]	QUIPS	Very small sample; sequencing limited to 3 matched pairs; fixation method, fracture classification, and interval from injury not reported	High
Waki et al. (2015) [19]	SYRCLE	Sequence generation, allocation concealment, random housing, and blinding of caregivers and outcome assessors not reported	Low to moderate
Xiao et al. (2024) [20]	SYRCLE	Randomization and blinding not reported; no human data	Low to moderate
Breulmann et al. (2023) [11]	AMSTAR-2	No registered protocol; no list of excluded studies with justification; impact of risk of bias on synthesis not assessed	Low confidence
Panteli et al. (2022) [2]	AMSTAR-2	No registered protocol; no list of excluded studies with justification; impact of risk of bias on synthesis not assessed	Low confidence

The two animal experiments appraised with SYRCLE were rated low to moderate risk. Waki et al. [19] employed a standardized model with 1:1 group allocation and serial sampling across six time points, which supports internal validity, but sequence generation, allocation concealment, random housing, blinding of caregivers and outcome assessors, and random outcome assessment were not reported. Xiao et al. [20] derived internal validity from convergent evidence across single-cell transcriptomics and three complementary genetic models whose phenotypes are mutually consistent, but likewise did not report randomization or blinding, and presented no human data. Both included systematic reviews [2,11] were rated as providing low confidence under AMSTAR-2, principally because neither reported a prospectively registered protocol, neither provided a list of excluded studies with justification, and neither formally assessed the impact of risk of bias on the synthesized results.

Long Non-coding RNAs

Two primary human studies directly examined long non-coding RNAs in non-union [17,18]. Du et al. studied 45 patients with long bone non-union and 45 matched controls with healed fractures, aged 20 to 50 years, with comparable baseline characteristics between groups [17]. Candidate selection was informed by the publicly available Gene Expression Omnibus dataset GSE213891, in which lnc_GABARAPL2 showed the largest upregulation of the three differentially expressed long non-coding RNAs identified, with a log2 fold change of 1.55. Serum lnc_GABARAPL2 was significantly higher in the non-union group (p < 0.001), and receiver operating characteristic analysis yielded an AUC of 0.922. On multivariable logistic regression adjusting for age, sex, American Society of Anesthesiologists classification, comorbidity index, injury severity score, Glasgow Coma Scale, abbreviated injury scale, concomitant injuries, open versus closed fracture, duration of hospital stay, in-hospital complications, and treatment strategy, lnc_GABARAPL2 remained independently associated with non-union (OR, 11.661; 95% CI, 3.794 to 35.840; p < 0.001). Overexpression in human bone marrow mesenchymal stromal cells suppressed alkaline phosphatase activity, RUNX2, collagen I, osteocalcin, and mineralization, and knockdown produced the converse effects. The long non-coding RNA acts by sequestering miR-302a-3p, with serum levels of the two inversely correlated (r = -0.798, p < 0.001) and direct binding confirmed by dual-luciferase reporter assay.

Two features of this study constrain the interpretation of its discrimination estimate. Serum was drawn preoperatively from patients already presenting for non-union surgery while the comparison group had already achieved union, so that the estimate distinguishes established non-union from achieved union rather than predicting which patients will subsequently fail, a point the authors themselves acknowledge in recommending validation within narrower post-fracture time windows. In addition, an AUC of 0.922 obtained in the same sample in which the marker was selected, without an external validation cohort, is expected to be optimistically biased. It is further notable that treatment strategy, comparing early total care with damage control orthopedics, was itself significantly associated with healing status within this cohort (p = 0.033).

Ouyang et al. compared fracture-site scar tissue obtained at operation from 10 patients with non-union and 14 patients with normal fracture healing, performing RNA sequencing on three pairs matched for sex, age, and fracture location with quantitative polymerase chain reaction validation across all 24 specimens [18]. Long non-coding RNA ENST00000563492 was downregulated in non-union tissue. Overexpression in bone marrow mesenchymal stromal cells increased alkaline phosphatase activity, mineralization, COL1A1, RUNX2, and osteocalcin, and promoted SMAD1 phosphorylation, while knockdown reversed these effects. Mechanistically, the transcript functions as a competing endogenous RNA for miR-205-5p, which targets CDH11 and vascular endothelial growth factor, thereby coupling osteogenesis through the CDH11 and beta-catenin axis with angiogenesis through vascular endothelial growth factor. Conditioned medium from overexpressing cells enhanced endothelial migration, invasion, and tube formation, and overexpression improved bone formation in both ectopic osteogenesis and femoral monocortical defect models in vivo. No discrimination metrics were reported.

These findings are supported by studies of long non-coding RNAs in osteogenesis outside the non-union context, in which long non-coding RNA-2271 promoted osteogenic differentiation of human bone marrow stem cells through regulation of RUNX2 promoter methylation [21], and long non-coding RNAs have been proposed as biomarkers regulating the osteogenic differentiation process in the management of bone defects [22]. Taken together, the two primary transcripts show opposite directions of expression in non-union but concordant functional effects on osteogenesis, which suggests that gain of function in an inhibitory long non-coding RNA and loss of function in a pro-osteogenic one may converge on impaired healing through complementary mechanisms.

MicroRNAs

Waki et al. created healing fractures and atrophic non-unions, the latter produced by periosteal cauterization, in the femora of 94 rats with 1:1 group allocation [19]. MicroRNAs were extracted from newly generated tissue at the fracture site at postfracture days 3, 7, 10, 14, 21, and 28. Microarray analysis with real-time polymerase chain reaction validation of day-14 samples demonstrated that five microRNAs were highly upregulated in non-union: miR-31a-3p, miR-31a-5p, miR-146a-5p, miR-146b-5p, and miR-223-3p. This peak corresponds to the reparative phase of healing in the rat, which suggests that dysregulation within this window may be particularly consequential for the eventual outcome. No discrimination metrics were reported, and the design is experimental rather than prognostic, so the study establishes biological association within a controlled model rather than predictive performance in patients. Profiling by the same group during normal fracture healing identified a distinct temporal signature, underscoring that the non-union profile is not simply a delayed version of the healing profile [23], and microRNAs have been proposed more broadly as orthobiologic agents for skeletal fractures on the basis of these expression patterns [24].

DNA Methylation

Xiao et al. investigated why skeletal repair fails in the setting of systemic inflammation [20]. Single-cell RNA sequencing identified skeletal progenitor cells as one of the principal lineages responding to elevated inflammation, with Rbpjk upregulated in fracture non-union. Using mice with constitutive activation of Ikk2 to model systemic inflammation, the authors validated the interaction between inflammation and Rbpjk specifically within progenitor cells, and subsequently identified Rbpjk as a direct target of Dnmt3b. Inflammation decreased Dnmt3b expression in progenitor cells, producing hypomethylation within the Rbpjk promoter region and consequent upregulation of Rbpjk, which impaired progenitor differentiation. Mice with loss of function of Dnmt3b phenotypically recapitulated the fracture repair defects observed in the inflammation model, whereas Dnmt3b transgenic mice alleviated those defects.

The direction of this mechanism merits explicit statement, because it inverts the intuition that promoter methylation, by silencing transcription, would be expected to drive a failure state. Methylation of the Rbpjk promoter is protective in this system, since it restrains a Notch effector whose overexpression blocks progenitor differentiation, and inflammation causes non-union by removing that methylation rather than by adding it. The therapeutic implication is accordingly restoration of Dnmt3b activity or targeted remethylation at this locus rather than administration of demethylating agents, which on this evidence would be expected to aggravate rather than rescue the defect. This reading is consistent with the supporting literature, in which conditional deletion of Dnmt3b in chondrocytes delayed endochondral ossification and impaired fracture repair [25], and Dnmt3b ablation impaired repair through upregulation of the Notch pathway [26]. The study contains no human validation cohort, and no genome-wide DNA methylation profiling of human non-union tissue has been reported.

Evidence From Included Systematic Reviews

Breulmann et al. synthesized 19 studies examining microRNAs in fracture non-union or delayed healing, alongside 82 studies addressing general fracture healing, and identified miR-31-5p, miR-221, and miR-451-5p as the most consistently associated microRNAs, while noting substantial heterogeneity in fracture models, sample collection timing, tissue sources, and measurement platforms and concluding that clinical utility remains unvalidated [11]. Panteli et al. synthesized 24 studies of human tissue molecular profiles in non-union and reported the presence of biologically active mesenchymal stromal cells within non-union tissue together with altered bone morphogenetic protein expression, with limited standardized epigenetic data [2]. Both reviews incorporate primary studies that are also cited directly in the present review, and in particular Waki et al. [19] is among the studies synthesized by Breulmann et al. [11]. The two bodies of evidence are therefore not independent, and the apparent convergence on miR-31 family members between a primary animal model and a systematic review is in part a restatement of the same underlying data rather than genuine external replication, a point we emphasize because earlier accounts of this literature, including our own, have treated that convergence as the most robust finding in the field.

Histone Modifications and Other Non-coding RNAs

No primary study examined histone modifications as markers of fracture non-union. Supporting studies confirm that histone acetylation and methylation regulate osteoblast and osteoclast differentiation [4,7], that histone deacetylases and acetyltransferases modulate bone formation and resorption, and that histone modifications respond to mechanical loading of the kind relevant to fracture-site biomechanics, with cross-talk between histone modifications and DNA methylation documented in bone loss [6]. None of this evidence extends to the non-union context. Similarly, no study examined circular RNAs, small nucleolar RNAs, or other emerging non-coding RNA classes in fracture non-union, despite their established roles in cellular differentiation and tissue homeostasis. These therefore represent entirely unexplored areas with considerable potential for future investigation.

Certainty of Evidence

Certainty of evidence, summarized by marker class in Table 3, was low for lnc_GABARAPL2 [17], downgraded for risk of bias arising from the case-control design and for imprecision reflecting a single study without external validation, and low for the miR-31 family [11,19] and for Rbpjk promoter methylation [20], downgraded for risk of bias and for indirectness given reliance on animal models and on overlapping secondary evidence. Certainty was very low for ENST00000563492 [18], for miR-146a-5p, miR-146b-5p, and miR-223-3p [19], and for miR-221 and miR-451-5p [11], in each case reflecting single-study evidence, absent effect estimates with variance measures, and indirectness of marker role. Publication bias could not be assessed formally, but underrepresentation of null findings is probable within a small candidate-marker literature, and no marker class was upgraded on any domain.

**Table 3:** Summary of Epigenetic Markers Associated With Fracture Non-union ALP: alkaline phosphatase; AUC: area under the receiver operating characteristic curve; CI: confidence interval; GRADE: Grading of Recommendations Assessment, Development and Evaluation; NF-κB: nuclear factor of kappa light-chain enhancer of activated B cells; OCN: osteocalcin; OR: odds ratio; VEGF: vascular endothelial growth factor

Marker	Direction in Non-union	Marker Role	Evidence Base	Discrimination Reported	GRADE Certainty	Mechanistic Basis
lnc_GABARAPL2 [17]	Upregulated (serum)	Diagnostic (case-control)	1 human study (N = 90)	AUC 0.922; OR 11.661 (95% CI, 3.794 to 35.840); not externally validated	Low	Sequesters miR-302a-3p; suppresses ALP, RUNX2, collagen I, and OCN
ENST00000563492 [18]	Downregulated (tissue)	Diagnostic	1 human study (N = 24)	Not reported	Very low	Competing endogenous RNA for miR-205-5p; CDH11 and beta-catenin; VEGF
miR-31a-5p and miR-31-5p [11,19]	Upregulated	Mechanistic and secondary	1 animal study; 1 systematic review (overlapping)	Not reported	Low	Inhibits osteoblast differentiation; targets RUNX2 and osterix
miR-146a-5p, miR-146b-5p, miR-223-3p [19]	Upregulated	Mechanistic	1 animal study	Not reported	Very low	NF-kB signaling; inflammatory regulation; macrophage polarization
miR-221, miR-451-5p [11]	Dysregulated	Secondary	1 systematic review	Not reported	Very low	Osteogenic and inflammatory pathway regulation
Rbpjk promoter methylation [20]	Hypomethylated, with Rbpjk upregulated	Mechanistic	1 animal study using multiple genetic models	Not reported	Low	Inflammation reduces Dnmt3b, causing promoter hypomethylation and Rbpjk upregulation; methylation is protective
Histone modifications	Not identified	Not applicable	No studies	Not applicable	Not applicable	Mechanistically relevant but unstudied in non-union
Circular and other non-coding RNAs	Not identified	Not applicable	No studies	Not applicable	Not applicable	Entirely unexplored

Discussion

This systematic review identified six studies directly addressing epigenetic markers in fracture non-union, comprising four primary studies [17-20] and two systematic reviews [2,11] whose constituent evidence partly overlaps. The most important finding is that the literature, although it now contains one marker with a reported discrimination estimate in an accessible compartment, does not yet establish that any epigenetic marker predicts future non-union. Serum lnc_GABARAPL2 is the most developed candidate, with an AUC of 0.922 and an adjusted OR of 11.661 in 90 patients, together with functional validation demonstrating suppression of osteogenic differentiation through sequestration of miR-302a-3p [17]. ENST00000563492 is downregulated in non-union tissue and promotes osteogenesis and angiogenesis through CDH11 and vascular endothelial growth factor [18]. Five microRNAs are upregulated at the reparative phase in a rat model [19], and the miR-31 family recurs in a systematic review that incorporates that same model [11]. Xiao et al. provide the most complete mechanistic account, demonstrating that inflammation reduces Dnmt3b and thereby hypomethylates and de-represses Rbpjk with consequent impairment of progenitor differentiation [20]. Histone modifications and non-canonical non-coding RNA classes remain entirely unexamined.

The presence of a single strong discrimination estimate should not be read as evidence of prospective predictive validity, and this distinction is the principal interpretive contribution of the present review. Du et al. drew serum preoperatively from patients already presenting for non-union surgery while the comparison group had already healed, so that the design establishes that lnc_GABARAPL2 distinguishes patients with established non-union from patients who united, which is a diagnostic contrast, rather than establishing that it identifies at the time of injury which patients will subsequently fail [17]. The estimate was moreover derived in the same sample in which the marker was selected and without external validation, and estimates obtained under those conditions are characteristically optimistic. The limitation applies more severely elsewhere, since Ouyang et al. sampled tissue at revision surgery [18], no discrimination metrics exist for any other marker, and no candidate has been examined in an independent cohort. Across the field, an epigenetic difference observed at a failed fracture site remains as compatible with being a consequence of the failed healing environment, including persistent inflammation, fibrous interposition, mechanical instability, and prior surgery, as with being its cause [2].

A second point concerns therapeutic direction, where the Rbpjk finding is liable to misreading and has, in our view, already been miscited. Because promoter methylation typically silences transcription and because non-union is a failure state, it is tempting to infer that hypermethylation drives non-union and that demethylating agents would therefore be beneficial. The data demonstrate the reverse [20]. Dnmt3b-dependent methylation of the Rbpjk promoter is required for normal repair, inflammation removes it, and restoration of Dnmt3b rescues healing, so that any translational program arising from this pathway should aim at restoring rather than removing methylation at this locus, a conclusion reinforced by independent demonstrations that loss of Dnmt3b impairs repair [25,26]. We state this explicitly because an inverted reading would point investigators and, ultimately, clinicians toward an intervention that the source evidence indicates would aggravate the defect.

Union is determined substantially by the mechanical environment, and the failure of this literature to account for that fact limits what any of its findings can support. Fracture pattern and anatomical site, energy of injury and soft-tissue status, open versus closed injury, infection, and above all the fixation strategy and the construct stability it produces are established determinants of healing [1]. Only Du et al. reported any treatment variable, and within that cohort the association between treatment strategy and healing status was itself statistically significant (p = 0.033), which demonstrates that management decisions carry independent prognostic weight in precisely the population used to estimate marker performance [17]. No included study reported AO/OTA classification or construct stability in a form permitting stratified or adjusted analysis. An epigenetic difference between non-union and healing cases therefore cannot be cleanly separated from the biological consequences of the mechanical construct that preceded it, and a marker elevated in non-union tissue may be reporting on instability rather than on any intrinsic biological predisposition. These variables require treatment as core covariates in future work rather than as descriptive detail, and prospective cohorts should prespecify stratification by anatomical site, AO/OTA classification, and fixation strategy, report construct stability and adherence to the fixation protocol, adjust for infection and open injury, and be powered for the resulting subgroups.

The strengths of this systematic review are that it applies appraisal instruments matched to the design of each included study rather than a single tool applied uniformly [14-16], that it classifies evidence explicitly by marker role, that it assesses certainty formally with GRADE, and that every quantitative finding was verified against the source full text rather than the abstract. That verification corrected several errors of fact carried by earlier accounts of this literature, including our own previous version, of which the most consequential was an inverted description of the Rbpjk methylation mechanism [20]. Despite these strengths, the review has several limitations. First, the search was performed in MEDLINE with supplementary retrieval from Google Scholar and a semantic tool, and Embase, Web of Science, and Scopus were not searched; since semantic retrieval does not yield a reproducible query in the sense required by PRISMA [12] and the majority of records were obtained by that route, eligible studies may have been missed and the six-study core evidence base should be regarded as a lower bound. Second, the protocol was not prospectively registered. Third, only articles published in English were included, posing a risk of language bias, and underrepresentation of null findings is probable within a small candidate-marker literature. Fourth, the included systematic reviews are not independent of the primary studies they contain, so that the effective evidence base is smaller than a count of six studies implies. Finally, at the level of the primary evidence, sample sizes were small, most notably the 24 specimens in Ouyang et al. with sequencing performed on three pairs [18]; sampling was case-control or cross-sectional and cannot establish temporal precedence; definitions of non-union and methods of radiographic ascertainment varied; blinding of outcome assessment was not reported; and no study developed or externally validated a clinical prediction model or assessed clinical utility, patient-reported outcomes, or cost-effectiveness.

The most valuable next study is a prospective cohort sampling serum at or near the index injury, before the outcome is known, with follow-up to a prespecified union endpoint. Serum lnc_GABARAPL2 is the obvious first candidate, since it is measurable in an accessible compartment, has a reported effect size with a confidence interval, and has performed well enough in a case-control setting to justify prospective evaluation [17]. Such a study should report discrimination with confidence intervals and calibration, follow the TRIPOD statement for model development and the STARD statement for accuracy reporting, prespecify the analysis plan, incorporate an external validation cohort, and stratify by fixation strategy and fracture characteristics. The decisive question, which has not yet been posed of any epigenetic marker in this field, is whether marker information adds discrimination beyond a baseline model comprising established clinical risk factors. Beyond that, genome-wide DNA methylation profiling and comprehensive small RNA sequencing in human non-union tissue, together with any primary investigation of histone modifications in the non-union context [4,6,7], would address complete evidentiary gaps, and the Rbpjk pathway identifies restoration of Dnmt3b activity as a candidate intervention for inflammation-associated non-union that warrants controlled preclinical evaluation before any clinical consideration [20,25,26].

## Conclusions

In conclusion, long non-coding RNAs and microRNAs are biologically plausible correlates of fracture non-union, with functional evidence linking them to dysregulated osteogenesis, and serum lnc_GABARAPL2 is the most developed candidate, distinguishing patients with established long bone non-union from healed controls with an AUC of 0.922 and an adjusted OR of 11.661 while acting mechanistically through sequestration of miR-302a-3p. Dnmt3b-dependent methylation of the Rbpjk promoter emerges as protective against inflammation-associated non-union, which identifies restoration rather than removal of methylation as the therapeutic direction at this locus. The evidence base nonetheless comprises only six studies of low to very low certainty; no study sampled human material before the healing outcome was known, the single discrimination estimate derives from a case-control design without external validation, and fixation strategy and fracture classification, which are dominant determinants of union, were essentially unreported. Histone modifications and non-canonical non-coding RNA classes remain wholly unexamined.

This review therefore identifies a credible candidate rather than a validated predictor, and establishing clinical utility will require prospective cohorts with early sampling, external validation, reporting of discrimination and calibration, demonstration of incremental value over established clinical risk factors, and stratification by fixation strategy. Until such studies exist, epigenetic markers in fracture non-union should be described as candidates under investigation rather than as prognostic tools.
